# How Can the COVID-19 Pandemic Lead to Positive Changes in Urology Residency?

**DOI:** 10.3389/fsurg.2020.563006

**Published:** 2020-11-24

**Authors:** Gian Maria Busetto, Francesco Del Giudice, Andrea Mari, Isabella Sperduti, Nicola Longo, Alessandro Antonelli, Maria Angela Cerruto, Elisabetta Costantini, Marco Carini, Andrea Minervini, Bernardo Rocco, Walter Artibani, Angelo Porreca, Francesco Porpiglia, Rocco Damiano, Marco De Sio, Davide Arcaniolo, Sebastiano Cimino, Giorgio Ivan Russo, Giuseppe Lucarelli, Pasquale Di Tonno, Paolo Gontero, Francesco Soria, Carlo Trombetta, Giovanni Liguori, Roberto Mario Scarpa, Rocco Papalia, Carlo Terrone, Marco Borghesi, Paolo Verze, Massimo Madonia, Antonello De Lisa, Pierluigi Bove, Giorgio Guazzoni, Giovanni Lughezzani, Marco Racioppi, Luca Di Gianfrancesco, Eugenio Brunocilla, Riccardo Schiavina, Claudio Simeone, Alessandro Veccia, Francesco Montorsi, Alberto Briganti, Fabrizio Dal Moro, Carlo Pavone, Vincenzo Serretta, Savino Mauro Di Stasi, Andrea Benedetto Galosi, Luigi Schips, Michele Marchioni, Emanuele Montanari, Giuseppe Carrieri, Luigi Cormio, Francesco Greco, Gennaro Musi, Martina Maggi, Simon L. Conti, Andrea Tubaro, Ettore De Berardinis, Alessandro Sciarra, Michele Gallucci, Vincenzo Mirone, Ottavio de Cobelli, Matteo Ferro

**Affiliations:** ^1^Sapienza Rome University Policlinico Umberto I, Rome, Italy; ^2^University of Florence, Florence, Italy; ^3^Istituto di Ricovero e Cura a Carattere Scientifico (IRCCS) Regina Elena National Cancer Institute, Rome, Italy; ^4^University of Naples Federico II, Naples, Italy; ^5^University of Verona, Verona, Italy; ^6^University of Perugia, Perugia, Italy; ^7^University of Modena, Modena, Italy; ^8^Abano Terme Policlinic, Abano Terme, Italy; ^9^University of Turin, Turin, Italy; ^10^University of Catanzaro, Catanzaro, Italy; ^11^University of Campania “L. Vanvitelli”, Caserta, Italy; ^12^University of Catania, Catania, Italy; ^13^University of Bari, Bari, Italy; ^14^University of Trieste, Trieste, Italy; ^15^University Campus Biomedico, Rome, Italy; ^16^University of Genoa, Genoa, Italy; ^17^University of Salerno, Salerno, Italy; ^18^University of Sassari, Sassari, Italy; ^19^University of Cagliari, Cagliari, Italy; ^20^University of Tor Vergata, Rome, Italy; ^21^Humanitas University, Milan, Italy; ^22^University Cattolica del Sacro Cuore, Rome, Italy; ^23^University of Bologna, Bologna, Italy; ^24^University of Brescia, Brescia, Italy; ^25^San Raffaele University, Milan, Italy; ^26^University of Udine, Udine, Italy; ^27^University of Palermo, Palermo, Italy; ^28^University of Ancona, Ancona, Italy; ^29^University of Chieti, Chieti, Italy; ^30^University of Milan, Milan, Italy; ^31^University of Foggia, Foggia, Italy; ^32^Humanitas University Gavazzeni, Bergamo, Italy; ^33^Department of Urology, IEO European Institute of Oncology IRCCS, Milan, Italy; ^34^Stanford University, Palo Alto, CA, United States; ^35^Sapienza Rome University Sant'Andrea Hospital, Rome, Italy; ^36^Department of Oncology and Haematology-Oncology, University of Milan, Milan, Italy

**Keywords:** urology, residency, residents, pandemic, COVID-19

## Abstract

The COVID-19 outbreak, in a few weeks, overloaded Italian hospitals, and the majority of medical procedures were postponed. During the pandemic, with hospital reorganization, clinical and learning activities performed by residents suffered a forced remodulation. The objective of this study is to investigate how urology training in Italy has been affected during the COVID-19 era. In this multi-academic study, we compared residents' training during the highest outbreak level with their previous activity. Overall 387 (67.1%) of the 577 Italian Urology residents participated in a 72-h anonymous online survey with 36 items sent via email. The main outcomes were clinical/surgical activities, social distancing, distance learning, and telemedicine. Clinical and learning activity was significantly reduced for the overall group, and after categorizing residents as those working only in COVID hospitals, both “junior” and “senior” residents, and those working in any of three geographical areas created (Italian regions were clustered in three major zones according to the prevalence of COVID-19). A significant decrease in outpatient activity, invasive diagnostic procedures, and endoscopic and major surgeries was reported. Through multivariate analysis, the specific year of residency has been found to be an independent predictor for all response modification. Being in zone 3 and zone 2 and having “senior” resident status were independent predictors associated with a lower reduction of the clinical and learning activity. Working in a COVID hospital and having “senior” resident status were independent predictors associated with higher reduction of the outpatient activity. Working in zone 3 and having “senior” resident status were independent predictors of lower and higher outpatient surgical activity, respectively. Working in a COVID hospital was an independent predictor associated with robotic surgical activity. The majority of residents reported that distance teaching and multidisciplinary virtual meetings are still not used, and 44.8% reported that their relationships with colleagues decreased. The COVID-19 pandemic presents an unprecedented challenge, including changes in the training and education of urology residents. The COVID era can offer an opportunity to balance and implement innovative solutions that can bridge the educational gap and can be part of future urology training.

## Introduction

The acute respiratory disease caused by coronavirus (SARS-CoV-2 or 2019-nCoV) and known as coronavirus disease 2019 (COVID-19) spread initially throughout China and from February 2020 in Europe and the USA (1).

After the COVID-19 outbreak, in <3 weeks, the virus led to overloaded hospitals in northern Italy, offering a glimpse of what countries face if they cannot slow the contagion. In particular, Intensive Care Units saw a rapid increase of admitted patients and saturated beds. As a result, the majority of nonurgent and non-oncological outpatients and surgical procedures have been postponed to facilitate COVID-19 patients assistance.

Also, urology departments required a full reorganization for both outpatients and surgical procedures, and most of them have been dedicated to the management of urological urgencies and interventions for cancer patients only (2, 3).

In Italy, residency programs are based on a minimum training program that needs to be accomplished in order to become a specialized doctor (4). A urology residency, one of the most challenging, is based on frontal lessons and clinical and surgical training based on outpatient, inpatient, and surgical activities. The required attendance is 5 years with a minimum of 38 h per week. During the COVID-19 pandemic and following hospital reorganization this program is suffering a forced changes. Since March 2020, clinical activities and, in particular, surgical procedures performed by residents have been decreasing, and it is difficult to predict the exact duration of the current situation.

In light of this, we aimed to investigate how urology training in Italy has been affected since the beginning of the COVID-19 pandemic and what changes have been embraced to overcome current limitations and constraints, including social distancing, distance learning, and telemedicine. In addition, we evaluated the use of personal protective equipment (PPE) among urology residents.

## Methods and Materials

### Study Design and Population

Our online survey was sent to all 577 Italian urology residents via email on April 9th, 2020. The population was stratified on the basis of the residency year (PGY1–3: junior residents; PGY4–5: senior residents); type of hospital (COVID vs. non-COVID hospital during the month of March 2020; a COVID hospital was defined as any health care center where COVID-19 patients were regularly treated); and geographical area on the basis of the number of COVID-19 cases (regions of Italy were clustered in three major zones: zone 1 included regions with ≥10,000 cases, zone 2 included regions with between 2,000 and 10,000 cases, and zone 3 included regions with ≤ 2000 cases, with the evaluation performed on the 5th of April 2020).

### Characteristics of the Survey

We emailed a 72-h anonymous online survey—the time frame in which the survey could be answered—featuring 36 items (Supplementary Figure 2). The survey was divided into four sections:

1. General information: academic or nonacademic hospital, the region of Italy, the year of residency, and the type of hospital;
2. Pre-COVID training information (October or November 2019): weekly business hours, weekly outpatient clinical activity, monthly invasive diagnostics, monthly minor surgeries, monthly endoscopic surgeries and monthly major surgeries divided between open or minimally invasive;
3. During COVID training information (March 2020)—same as pre-COVID training information;
4. Other information related to COVID period—use of distance teaching, telemedicine, relation with colleagues and PPE availability/usage.

The survey was conducted in Italian according to the Checklist for Reporting Results of Internet E-Surveys (5). Four Italian urology opinion leaders reviewed the quality of the survey. Usability and technical functionality were checked before administering the questionnaire. The last question of the survey (Is this the first time you have filled in this questionnaire?) was inserted to check the reliability of the numbers and to avoid having people respond twice or more often.

The survey was distributed by contacting directors of all Italian residency programs. In addition, “Senato degli Specializzandi,” an association that includes the majority of Italian residents, has been asked to share the survey.

### Objectives of the Study

The objective of this study was to compare residents' clinical and surgical activities in the month of March 2020 (the highest level of COVID-19 the outbreak in Italy) with their activity during a non-COVID-19 period (October 2019 or November 2019). The role of distance teaching tools in residents' education, the use of telehealth medicine at academic centers, and the impact on social relationships among colleagues were also evaluated.

### Statistical Analysis

A descriptive analysis of the response of each of the questions of the survey was carried out. The association between variables was tested by the Pearson chi-square test or the Fisher exact test.

A comparison of the responses given regarding the pre-COVID and during-COVID training information was performed using the nonparametric McNemar test.

The odds ratio (OR) and 95% confidence interval (95% CI) were estimated for each variable. Statistical significance was set as *p* ≤ 0.05. The following variables were considered: zone, COVID hospital, and year of residency. Variables that were statistically significant in the univariate analysis were used in the multivariate analysis. A multivariate logistic regression model was developed using stepwise regression (forward selection) to compare the predictive power of different factors. The limit on testing a variable and removing it were *p* = 0.10 and *p* = 0.15, respectively.

A multiple correspondence analysis (MCA) was performed as a descriptive technique designed to analyze simple two-way and multiple-way tables. MCA was used to evaluate the possible relationships among all the variables and to identify specific profiles (6). Associations between features are represented graphically in MCA, providing a graphic representation of the statistical relationships among distinct features, with the position of each being exclusively informative. This representation aims to visualize the similarities and/or differences in the profiles simultaneously, identifying those dimensions that contain most of the data variability. The position of the points in the MCA graph is also informative. Categories that plot close to each other will be significantly related statistically and have patterns of relative frequencies. This association is also valuable statistically when the points are located far from the origin of the graph and represents a mean, uninformative profile.

The RStudio graphical interface v.0.98 for R software environment v.3.0.2 were used for all analyses.

## Results

### Baseline Characteristics

**Figure 1:**
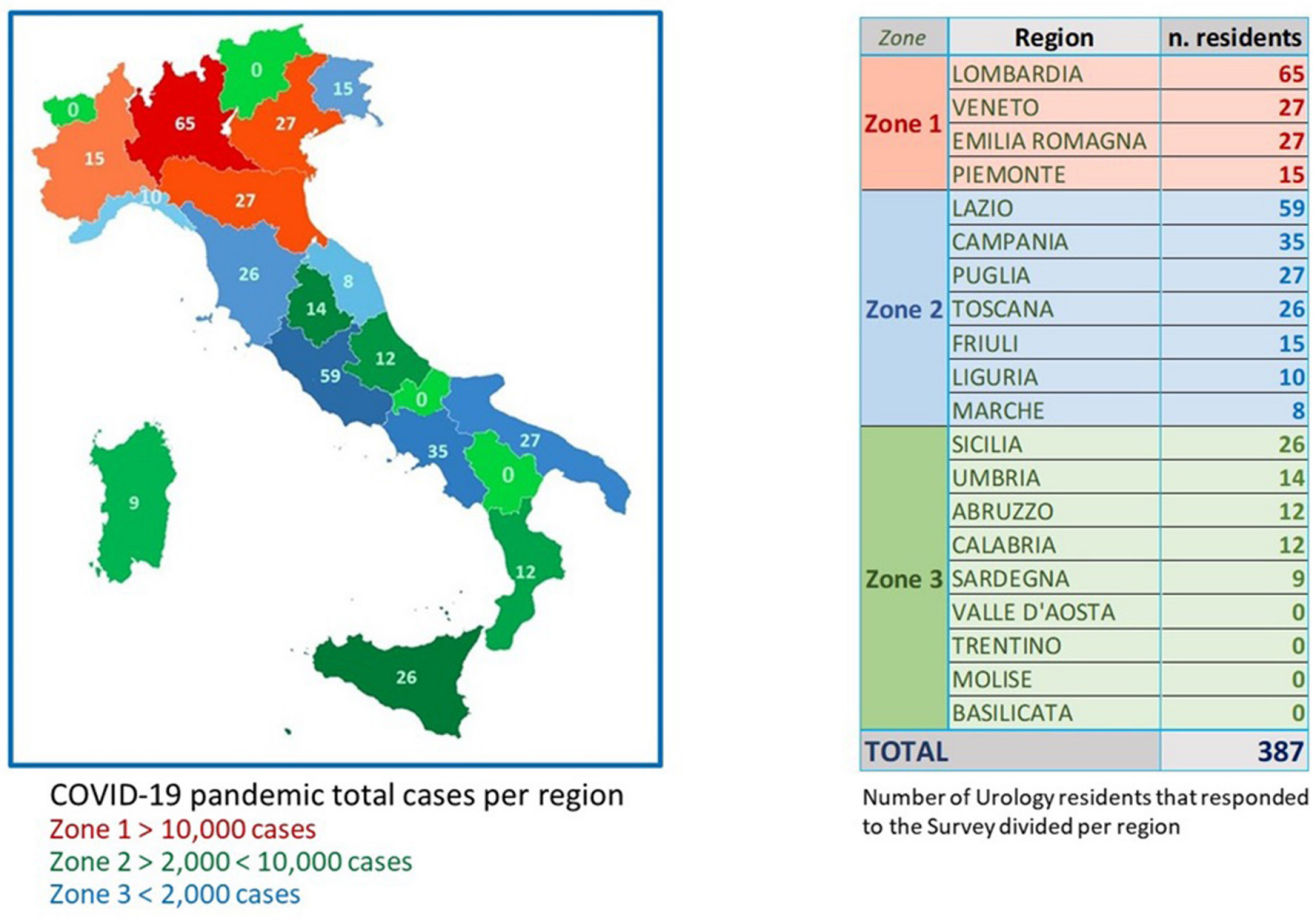
Topographical distribution of urology residents who completed our survey in relation to the three geographical zones defined on the basis of COVID-19 cases.

Overall, 387 (67.1%) of the 577 Italian urology residents completed the survey. A graphic representation of the regions of the hospitals of origin is depicted in Figure 1. Of these, 24.1, 39.2, and 36.7% were PGY1, PGY2-3, and PGY4-5 residents. Overall, 85.1% of them were working in a COVID hospital at the moment of completing the survey. During data collection no complications have been reported.

### Comparison Between Periods

**Figure 2:**
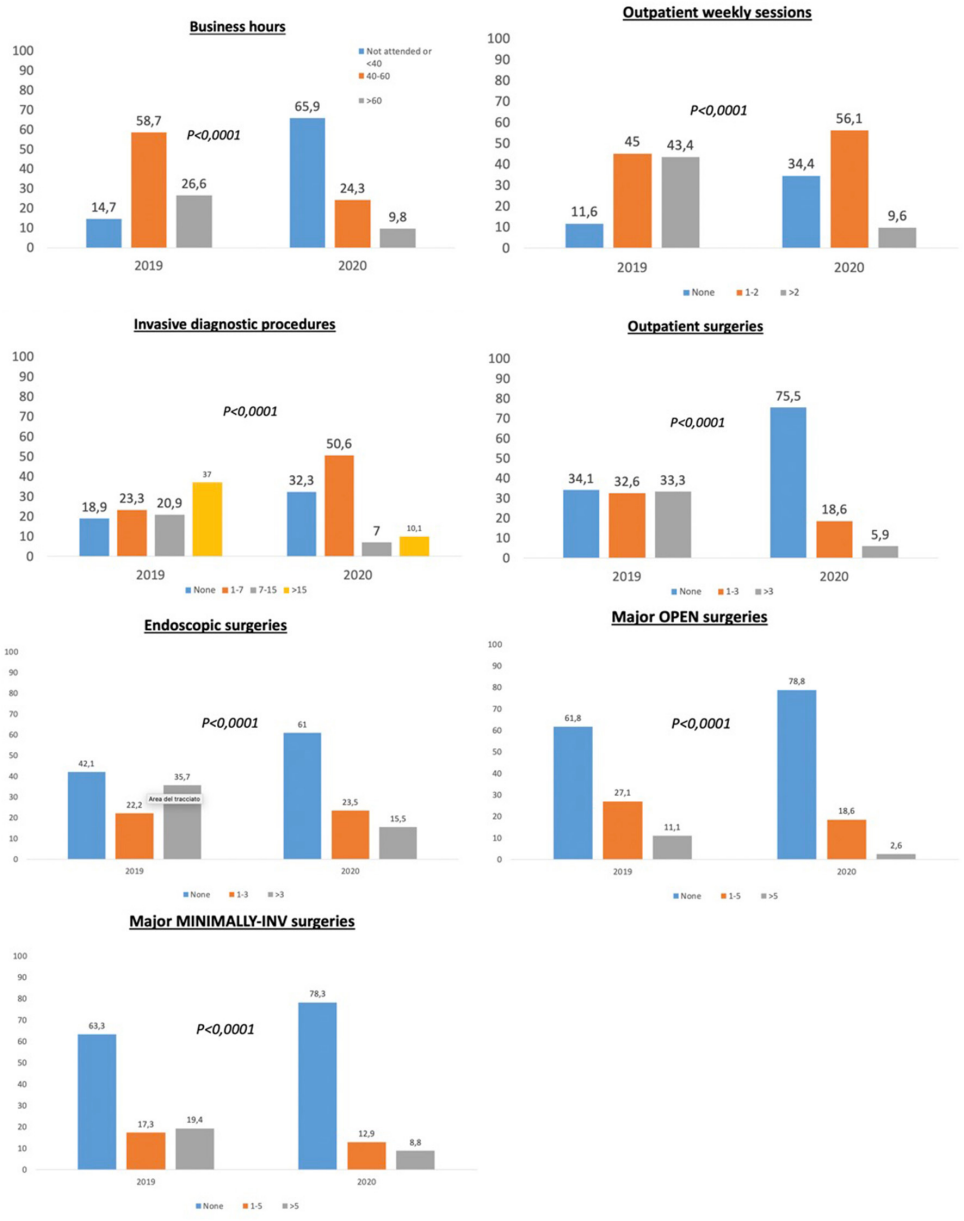
Histograms comparing different survey items between March 2020 (the highest outbreak level in Italy) and October or November 2019 (non-COVID-19 period), overall population.

Clinical and learning activity at the hospital was significantly reduced for the overall group (*p* < 0.0001), for those working only in COVID hospitals (*p* < 0.0001), for both “junior” and “senior” residents (both *p* < 0.0001) and for those working in any of the three geographical areas (all *p* < 0.0001). A comprehensive histogram overview concerning the cumulative response modification between pre- and COVID period for all items addressed in the survey is represented in Figure 2.

A significant decrease in outpatient and invasive diagnostic procedures was reported in the overall population and after categorizing for COVID hospital, year of residency, and geographical area (all *p* < 0.0001). Outpatient surgical activities significantly decreased in the overall group (*p* < 0.0001), for residents working only in COVID hospitals (*p* < 0.0001), for both “junior” and “senior” residents (both *p* < 0.0001) and for those working in all of the three geographical areas (all *p* < 0.0001), but it was not significantly reduced for those working in non-COVID hospitals (*p* = 0.23).

**Figure 3:**
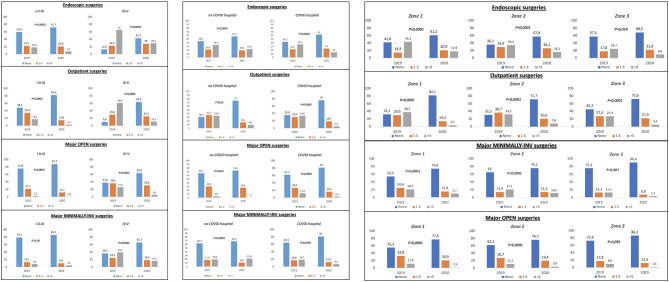
Histograms comparing surgical survey items (a: endoscopic, b: outpatient, c: major-open, and d: major-minimally invasive) between March 2020 (the highest outbreak level in Italy) and October or November 2019 (non-COVID-19 period) - stratification on the basis of the year of residency (I-II-III vs. IV-V), hospital kind (COVID or NO-COVID), geographical zones with different number of COVID-19 positive cases (regions of Italy were clustered in three major zones: zone 1 included regions with >10,000 cases, zone 2 included regions with between 2,000 and 10,000 cases, zone 3 included regions with <2,000 cases).

Endoscopic activity significantly decreased for the overall group and for the subgroups considered. Open and minimally invasive major surgical activity significantly decreased in the overall group (*p* < 0.0001), for residents working in either COVID or non-COVID hospitals (*p* < 0.0001), for both “junior” and “senior” residents (both *p* < 0.0001) and for those working in the zone 1 and 2 (all *p* < 0.0001). Histograms focusing on the differences among the sole surgical activities categorized according to geographic area, residency year, and COVID or non-COVID hospital are presented in Figure 3 while Supplementary Figures 3–5 contain all the outcomes concerning the study population from the full items addressed in the survey.

### Predictors of Reduced Activity

Uni- and multivariate logistic regression outcomes are presented in Table 1. Year of residency (“senior” vs. “junior” status) has been consistently found to be an independent predictor for all response modification during the COVID period. Moreover, zone 3 (OR 0.53, 95% CI 0.17–0.92, *p* < 0.0001) and zone 2 (OR 0.55, 95% CI 0.33–0.91, *p* = 0.02) compared to zone 1 of origin, and “senior” resident status (OR 0.44, 95% CI 0.29–0.69, *p* < 0.0001) were independent predictors associated with a lower reduction of the clinical and learning activity. Working in a COVID hospital (OR 1.87, 95%CI 1.05–3.35, *p* = 0.04) and “senior” resident status (OR 2.02, 95% CI 1.30–3.14, *p* = 0.002) were independent predictors associated with higher reduction of the outpatient activity. Working in zone 3 (OR 0.49, 95%CI 0.27–0.89, *p* = 0.02) compared to zone 1 and “senior” resident status (OR 3.67, 95%CI 2.29–5.88, *p* < 0.0001) were independent predictors associated with lower and higher outpatient surgical activity, respectively. Working in a COVID hospital (OR 4.64, 95% CI 2.90–7.44, *p* < 0.0001) was an independent predictor associated with robotic surgical activity as assistant (Table 1).

**Table 1:** Univariate and multivariate analysis. Impact of the year of residency, hospital type, and geographic zone on the modification of urology residents' clinical and learning activities during the COVID-19 pandemic.

**Variable**		**Univariate analysis**		**Multivariate analysis**	
		**OR (CI 95%)**	***p* value**	**OR (CI 95%)**	***p* value**
Modification - business hours/week	Zone	**–**	**0.003**	**–**	**0.001**
	Zone 2 vs. Zone 1	**0.569 (0.349–0.927)**	**0.024**	**0.551 (0.335–0.907)**	**0.019**
	Zone 3 vs. Zone 1	**0.363 (0.200–0.662)**	**0.001**	**0.320 (0.173–0.592)**	**<0.0001**
	Covid Hospital yes vs. no	1.060 (0.590**–**1.907)	0.845		
	PGY IV-V vs. I-II-III	**0.484 (0.316–0.743)**	**0.001**	**0.443 (0.285–0.688)**	**<0.0001**
Modification - outpatient clinics	Zone	**–**	0.437		
	Zone 2 vs. Zone 1	1.331 (0.847**–**2.091)	0.215		
	Zone 3 vs. Zone 1	1.076 (0.607**–**1.906)	0.803		
	Covid Hospital yes vs. no	**1.798 (1.015–3.186)**	**0.044**	**1.870 (1.046–3.345)**	**0.035**
	PGY IV-V vs. I-II-III	**1.915 (1.248–2.939)**	**0.003**	**2.021 (1.301–3.140)**	**0.002**
Modification - invasive diagnostic procedures	Zone	**–**	0.112		
	Zone 2 vs. Zone 1	0.819 (0.507**–**1.321)	0.413		
	Zone 3 vs. Zone 1	0.534 (0.296**–**0.963)	0.037		
	Covid Hospital yes vs. no	1.224 (0.683**–**2.194)	0.497		
	PGY IV-V vs. I-II-III	2.099 (1.331**–**3.311)	0.001	**2.099 (1.331–3.311)**	**0.001**
Modification - outpatient surgeries	Zone	**–**	**0.007**	**–**	**0.020**
	Zone 2 vs. Zone 1	1.061 (0.669**–**1.685)	0.801	1.084 (0.669**–**1.755)	0.743
	Zone 3 vs. Zone 1	**0.454 (0.254–0.810**)	**0.008**	**0.488 (0.266–0.894)**	**0.020**
	Covid Hospital yes vs. no	0.880 (0.493**–**1.572)	0.666		
	PGY IV-V vs. I-II-III	**3.765 (2.361–6.004)**	**<0.0001**	**3.674 (2.294–5.884)**	**<0.0001**
Modification - endoscopic surgeries	Zone	**–**	0.200		
	Zone 2 vs. Zone 1	1.136 (0.726**–**1.778)	0.578		
	Zone 3 vs. Zone 1	0.682 (0.380**–**1.222)	0.198		
	Covid Hospital yes vs. no	1.444 (0.805**–**2.588)	0.218		
	PGY IV-V vs. I-II-III	2.170 (1.425**–**3.303)	<0.0001	**2.170 (1.425–3.303)**	**<0.0001**
Modification - open major surgeries	Zone	**–**	0.472		
	Zone 2 vs. Zone 1	0.763 (0.475**–**1.225)	0.263		
	Zone 3 vs. Zone 1	0.748 (0.406**–**1.379)	0.352		
	Covid Hospital yes vs. no	1.233 (0.660**–**2.302)	0.512		
	PGY IV-V vs. I-II-III	3.219 (2.066**–**5.015)	<0.0001	**3.219 (2.066–5.015)**	**<0.0001**
Modification - minimally invasive surgeries	Zone	**–**	**0.026**	**–**	**0.037**
	Zone 2 vs. Zone 1	**0.559 (0.344–0.906)**	**0.018**	**0.528 (0.315–0.884)**	**0.015**
	Zone 3 vs. Zone 1	**0.494 (0.259–0.942)**	**0.032**	**0.546 (0.275–1.081)**	**0.08**
	Covid Hospital yes vs. no	1.115 (0.589**–**2.112)	0.739		
	PGY IV-V vs. I-II-III	**4.609 (2.896–7.335)**	**<0.0001**	**4.643 (2.898–7.438)**	**<0.0001**

*Bold values indicates the statistically significant values*.

**Figure 4:**
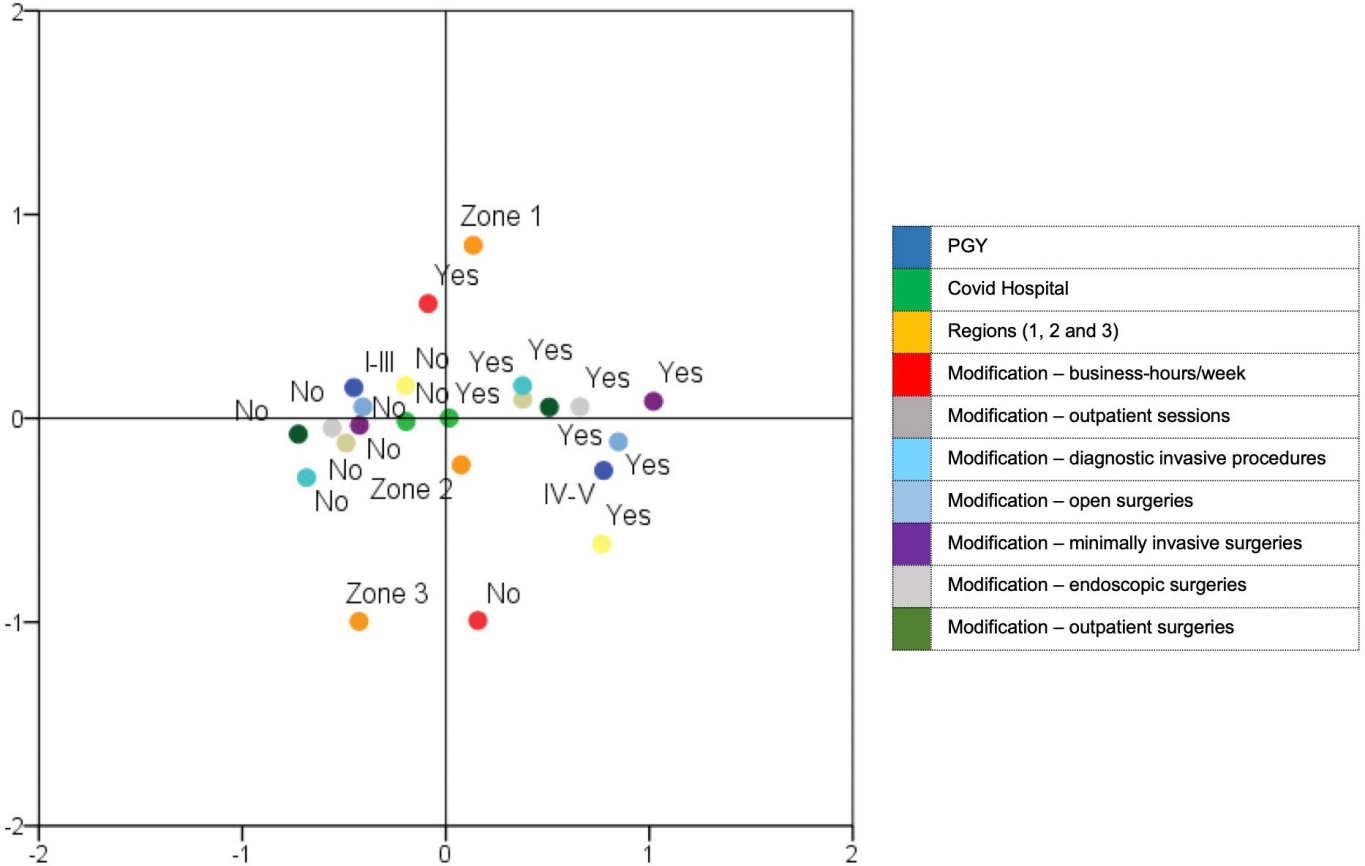
Multiple correspondence analysis (MCA) indicating interrelationships among parameters considered in order to evaluate the reduction of residents' activities clustered into phenotypic subtypes.

The MCA depicted in Figure 4 revealed the complex interrelationships among the several parameters considered in order to evaluate the reduction of residents' activity clustered into phenotypic subtypes. In detail, along the first axis, the test demonstrates the contrast between zone 1 and zone 2 and, in particular, three that are far from the origin and diagonally opposite, determining a different response to the survey. Besides, the second axis clearly differentiates the first triennium and the last biennium of activity showing that these two groups differently correlated with similar responses to the survey.

### Distance Teaching, Social Relations, and Use of PPE

In total, 52.9% of the residents reported that a distance teaching tools for the residents' education had never been used. However, 12.9 and 33.2% of the residents reported that the use of distance teaching tools for any kind of activities “increased” and “significantly increased,” respectively. Moreover, 61.1% of them reported that no multidisciplinary virtual meeting was performed at their hospital during March 2020. Compared to the past period, 44.8% of the residents reported that the relationship with their colleagues (residents, fellows, tutors, staff) decreased. Finally, Supplementary Figure 1 depicts the availability and usage of PPE.

A multivariate analysis found that working in a COVID hospital (OR 0.51, 95% CI 0.28–0.93, *p* = 0.03) was an independent predictor of the reduction of relationships with their colleagues, while working in zone 3 (OR 1.82, 95% CI 1.00–3.35, *p* = 0.05; comprehensive *p* = 0.005) was an independent predictor of the increase of relationship with their colleagues (Table 2).

**Table 2:** Univariate and multivariate analysis. Impact of the year of residency, hospital type, and geographic zones on the modification of urology residents' distance learning, telehealth, and relationships with colleagues during the COVID-19 pandemic.

**Variable**		**Univariate analysis**		**Multivariate analysis**	
		**OR (CI 95%)**	***p* value**	**OR (CI 95%)**	***p* value**
System of distance teaching (distance lesson, video conferencing, chat, etc.)^a^	Zone		0.527		
	Zone 2 vs. Zone 1	0.824 (0.527–1.290)	0.398		
	Zone 3 vs. Zone 1	0.735 (0.414–1.304)	0.293		
	Covid Hospital yes vs. no	1.555 (0.871–2.775)	0.135		
	PGY IV-V vs. I-II-III	1.029 (0.681–1.555)	0.893		
Clinical consultation or cases discussion with telematic meetings^b^	Zone	–	0.305		
	Zone 2 vs. Zone 1	1.169 (0.742–1.842)	0.502		
	Zone 3 vs. Zone 1	0.749 (0.411–1.362)	0.343		
	Covid Hospital yes vs. no	0.822 (0.464–1.457)	0.503		
	PGY IV-V vs. I-II-III	1.493 (0.981–2.271)	0.061		
Multidisciplinary meeting and/or consultations with telematic systems^c^	Zone	–	0.902		
	Zone 2 vs. Zone 1	0.894 (0.550–1.453)	0.652		
	Zone 3 vs. Zone 1	0.925 (0.493–1.733)	0.807		
	Covid Hospital yes vs. no	1.229 (0.667–2.264)	0.508		
	PGY IV-V vs. I-II-III	1.050 (0.668–1.652)	0.833		
Relationship with colleagues^d^	Zone	–	**0.004**	–	0.005
	Zone 2 vs. Zone 1	0.724 (0.462–1.136)	0.160	0.699 (0.441–1.108)	0.127
	Zone 3 vs. Zone 1	**1.929 (1.060**–**3.512)**	**0.032**	**1.828 (0.996**–**3.357)**	**0.052**
	Covid Hospital yes vs. no	**0.517 (0.285**–**0.937**)	**0.05**	**0.510 (0.278**–**0.934)**	**0.029**
	PGY IV-V vs. I-II-III	**0.664 (0.438-1.007)**	**0.03**	0.688 (0.447–1.060)	0.09

a *never vs. ≥ 1 time*.

b *no vs. yes*.

c *did not vary/diminished vs. increased*.

d *diminished vs. did not vary/increased. Bold values indicates the statistically significant values*.

## Discussion

This population was well representative of Italian urology residents' distribution in the different regions of the country (Figure 1).

An analysis of the overall results shows that all the parameters evaluated in the clinical practice section of the survey showed a global reduction of the residents' clinical activity compared to the pre-COVID era. In fact, the majority of residents worked fewer hours, attended fewer outpatient clinics, performed a lower number of diagnostic procedures and outpatient surgeries, and were involved to a lesser extent in major surgeries.

Interestingly, when data were categorized in accordance with type of hospital (COVID or non-COVID), we could not find a significant reduction of clinical/learning activity and of outpatients' surgery. This was most likely due to an activity that has been less affected in all the hospitals not dealing with COVID patients, where nonurgent and elective surgery was maintained. This was confirmed by analyzing the attendance of residents during endoscopic urological interventions.

Nevertheless, multivariate analysis showed that being in zone 2 and 3 and having senior resident status were associated with a lower reduction of clinical/learning activity and outpatient surgical activity, while working in a COVID hospital and having senior resident status were predictors associated with a higher reduction of the outpatient activity. These data confirm that COVID hospitals and zones with a higher number of SARS-CoV-2 cases are more involved in all clinical activity that could be deferred.

The multiple correspondence analysis indicates that residents working in zone 1 have been affected by a bigger change in their activity compared with those in zone 2 and an even a bigger change with zone 3.

On the other hand, it should be noted that distance teaching and telemedicine are still far from being considered a daily routine (7): even during the social distancing era, only 47.1% of residents had access to telematics training, and only a 38.9% attended a multidisciplinary telematics meeting.

Finally, the use of PPE reflects the actual shortage; surgical masks and gloves are the only equipment widely used with 89.8 and 72.5% of residents who are wearing them, respectively, outside an OR context. Generally, residents saw a reduction of relationships with their colleagues. Interestingly, this was higher overall in COVID hospitals compared with non-COVID hospitals. However, a significant increase in relationships with colleagues was noted in high-risk zones compared to low-risk zones. This apparent contradiction could be related to the fact that in the areas with very high death rates, support among colleagues increased to cope with the catastrophic situation that some hospitals were living through. Indeed, the pandemic will have meaningful psychological consequences on residents and on health care workers generally. Further studies are needed to carefully analyze in detail these aspects with particular attention to the risks of anxiety, depression, burn-out and all stressful consequences (8).

The health care system in Italy is a regionally based national health service that provides universal coverage. The last assessment by the World Health Organization (WHO) of health systems was performed in 2000, and the Italian health care system was the second best, after France. Even the most recent evaluation confirmed that the Italian health care system is the third best in the world (9, 10). Urology residency programs, part of this system, are among the most challenging and are continuously changing over time. The unprecedented COVID-19 pandemic had a very serious impact on residents' clinical and learning activities, and their training is suffering a dramatic decrease in quantity and quality. A recent survey reported that more than 75% of Italian urology residents used to work 50 or more hours per week (4), while in our survey we reported a strong decrease to 30% more or less. The quality of training, as well, is affected because senior urologists are more and more dedicated to best practices, lower surgical complications, and best ward management. Small surgeries and elective endoscopic procedures in which residents are more involved are those registering a higher decrease and this offers less opportunity to all residents to improve their skills (11). Recent Italian experience in the COVID-19 era, based on a survey with 25 items sent to urology residents, suggests a severe reduction or complete suppression of clinical and surgical activity. The proportion of residents that experienced a severe/complete reduction of training ranged from 41.1% to 81.2% (12).

The pandemic could be even the cause of different diagnostic and therapeutic strategies for minimizing the potential exposure of patients to hospitals, postponing low-risk surgeries, and delaying or reconsidering certain therapies (13). The risks and benefits should take into account the need for the patient to avoid worse disease management (14, 15). For example, some authors are proposing the Vesical Imaging-Reporting and Data System or circulating tumor cells as reliable alternative diagnostic tools to aid in risk categorization and to correctly diagnose and follow up on bladder cancer patients (16, 17). The impact for residents, once again, could be high and require a complete reorganization of their activity (18).

Looking toward the future, we should start thinking of different ways to provide adequate training in urology, and we should consider virtual learning platforms, for example, using web platforms such as Google Classroom, Google Meet, Zoom, or Webex (19–21). Although there is no substitute for hands-on learning through operating experience and direct patient care, surgical skills could be implemented even outside the OR using surgical simulators, such as surgical skill laboratories, cadaveric dissection and procedural training, computer-based virtual reality training, and endoscopic surgery simulation (22). Porpiglia et al. suggested an online dedicated platform offering videos of lessons and surgical procedures or webinar meetings. Social media, podcasts and blogs could be another tool to create a network to implement our knowledge (11, 12).

Such “cognitive training” might allow users to rehearse a procedure without carrying it out, offering a relative advantage while not necessitating electronic resources or particular costs or fees. This cognitive-driven approach has been demonstrated to be significantly productive with regard to other different fields, such as aviation, sports, and musical activities; while research is still limited, although it is proposed for surgical education.

In their comprehensive literature review of mental training in surgical education, Davison et al. focused on the act of performing motor tasks in the “mind's eye” and on the potential for training outside the operating room. The authors found that the majority of research studies reported mental training to be useful. Even if the cumulative level of evidence from the analysis was still relatively low, lacking standardized methodology and acknowledging a small sample size, the majority of the studies demonstrated a significant efficacy and impact, especially among the more experienced surgeons (23). Among urology training facilities, the use of simulators with the aim of reproducing a real-world process or system over time has been developed recently. Nevertheless, the main limitations of these approaches are the continuously stressful conditions surgeons have to face during any kind of procedures that can cause relevant variation in real-life surgical outcomes. In order to keep surgery safe, “nontechnical skills” training could be a part of any simulation-based training (24). An interesting trial conducted of 59 medical students who have been randomized in three groups: control-simulation training only, flashcards cognitive training, and mental imagery cognitive training. To evaluate skills improvement in endourology, subjects were tested with the URO Mentor performance report and a quantitative survey. Results showed that the role of cognitive training for the acquisition of surgical skills is minimal and that no form of cognitive training was superior to another (25).

Our analysis has several strengths, but there are some limitations that should be underscored: we evaluated only the situation in Italy, and we did not extend the survey to other European countries; the web-based system could leave out certain clinical and learning aspects while an objective assessment could not be reached because responders offered their personal judgment, not always reflecting real life. Finally, 67.1% of subjects who completed the survey during the 72 h allowed could be considered not fully representative of all urology residents.

## Conclusion

The COVID-19 pandemic presents an unprecedented challenge for our health system, including the training and education of urology residents. The dramatic change in residents' daily routines is reflected in a decrease in all clinical and learning activities. The COVID era can offer an opportunity to balance and implement innovative solutions that can bridge the educational gap and can be part of future urology training.

## Data Availability Statement

The raw data supporting the conclusions of this article will be made available by the authors, without undue reservation.

## Ethics Statement

The studies involving human participants were reviewed and approved by Department of Urology internal Ethical Committee, Sapienza University of Rome. The patients/participants provided their written informed consent to participate in this study.

## Author Contributions

GB, MF, and AS: study concept and design. NL, AA, MCe, EC, MCa, AMi, BR, WA, AP, FP, RD, MD, DA, SCi, GR, GLuc, GLi, GLug, PD, PG, FS, CTr, RSca, RP, CTe, MB, PV, MMad, AD, PB, GG, MR, LD, EB, RSch, CS, AV, FM, AB, FDa, CP, VS, SD, AG, LS, MMar, EM, GC, LC, FG, GM, AT, MG, VM, ED, and OC: acquisistion of data. FDe, AMa, GB, MF, SCo, and MMag: analysis and interpretation of data. IS: statistical analysis. AS, ED, and OC: supervision. All authors contributed to the article and approved the submitted version.

## Conflict of Interest

The authors declare that the research was conducted in the absence of any commercial or financial relationships that could be construed as a potential conflict of interest. The reviewer SL declared a shared affiliation with several of the authors to the handling editor at the time of review.

## Supplementary Material

Supplementary Figure 1 Use of personal protective equipment (PPE) by urology residents.

Supplementary Figure 2 The 36-item survey with possible answers.

Supplementary Figure 3 Histograms comparing different survey items between March 2020 (the highest outbreak level in Italy) and October or November 2019 (the non-COVID-19 period) categorized on the basis of the type of hospital (non-COVID or COVID hospital).

Supplementary Figure 4 Histograms comparing different survey items between March 2020 (the highest outbreak level in Italy) and October or November 2019 (the non-COVID-19 period), categorized on the basis of geographical zones with different numbers of COVID-19 cases (regions of Italy were clustered in three major zones: zone 1 included regions with ≥10,000 cases, zone 2 included regions with between 2,000 and 10,000 cases, and zone 3 included regions with ≤ 2,000 cases).

Supplementary Figure 5 Histograms comparing different survey items between March 2020 (the highest outbreak level in Italy) and October or November 2019 (the non-COVID-19 period), categorized on the basis of the year of residency (I-II-III vs. IV-V).
