# Supplementary figures and images for: How Can the COVID-19 Pandemic Lead to Positive Changes in Urology Residency?
Source: Front Surg. 2020 Nov 24;7:563006. doi: 10.3389/fsurg.2020.563006 (PMC7732553; doi:10.3389/fsurg.2020.563006)

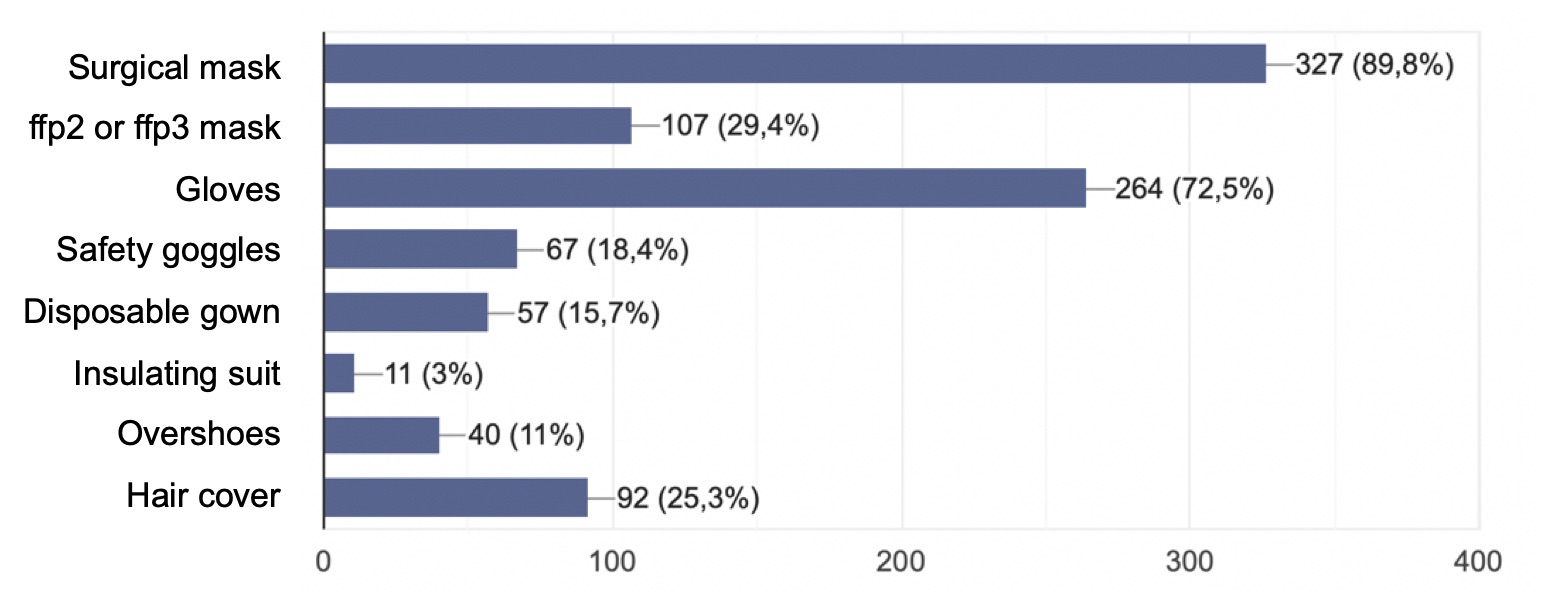

Supplement: Supplementary Figure 1 — Use of personal protective equipment (PPE) by urology residents. [file Image_1.JPEG]

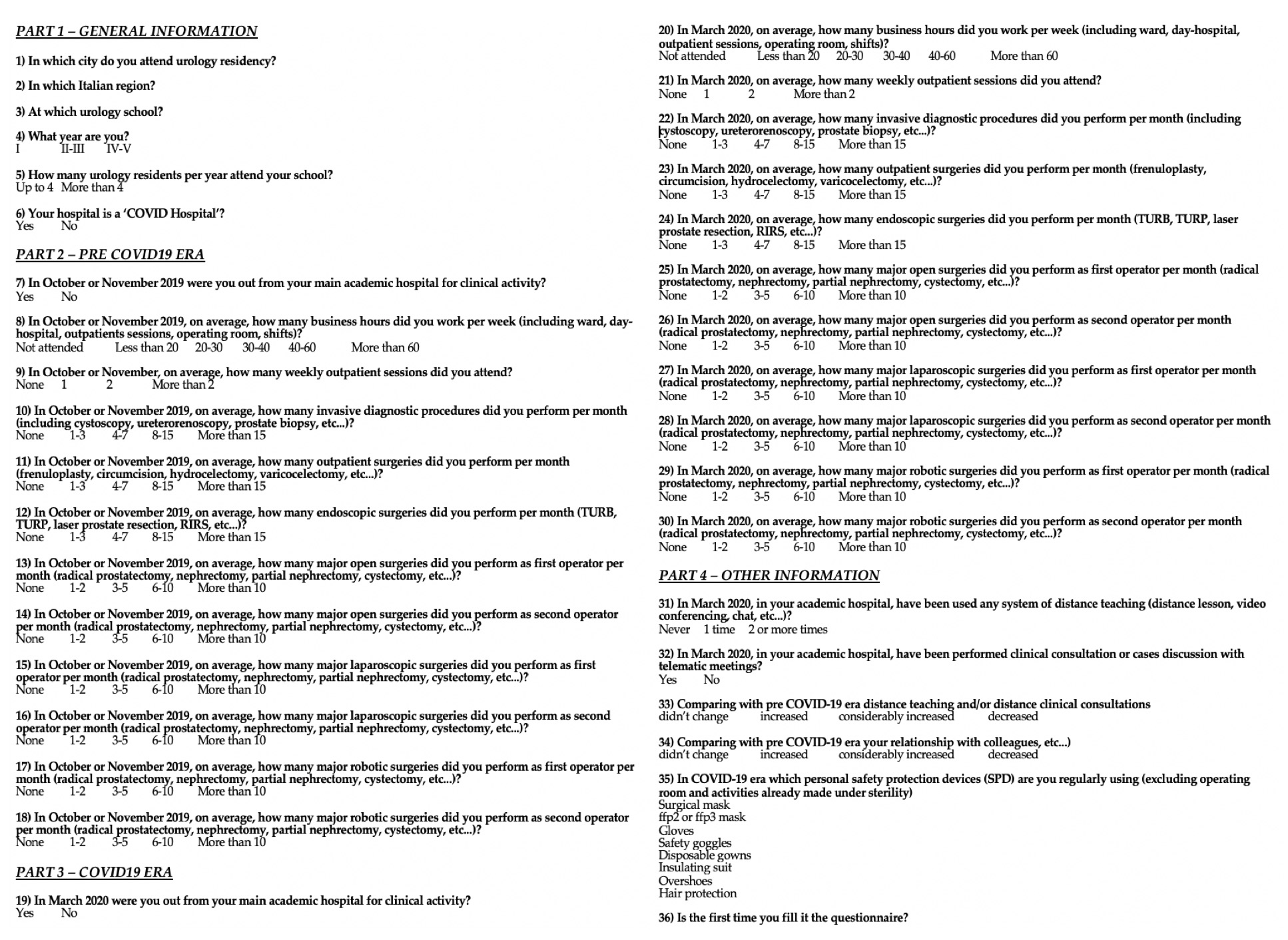

Supplement: Supplementary Figure 2 — The 36-item survey with possible answers. [file Image_2.JPEG]

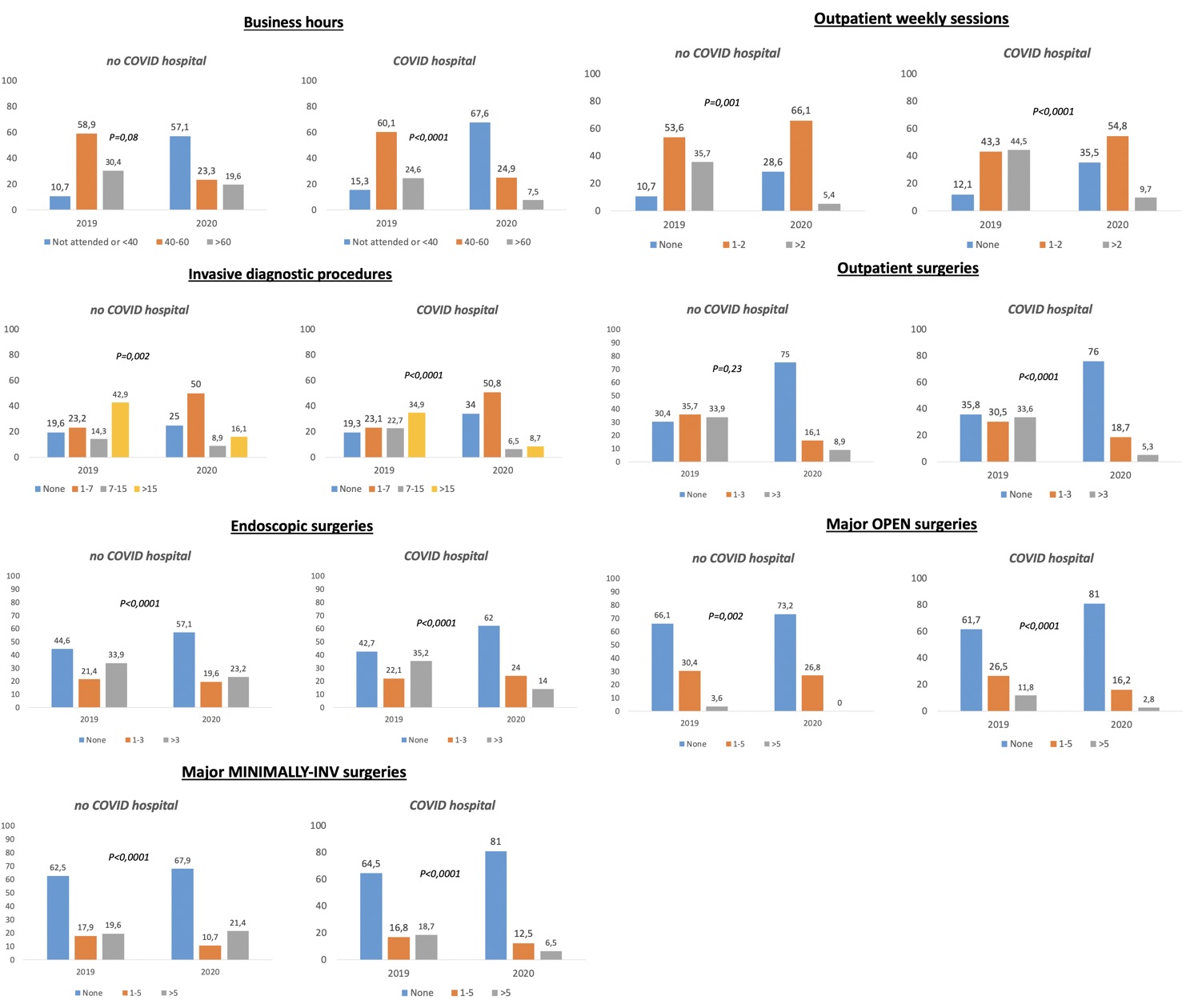

Supplement: Supplementary Figure 3 — Histograms comparing different survey items between March 2020 (the highest outbreak level in Italy) and October or November 2019 (the non-COVID-19 period) categorized on the basis of the type of hospital (non-COVID or COVID hospital). [file Image_3.JPEG]

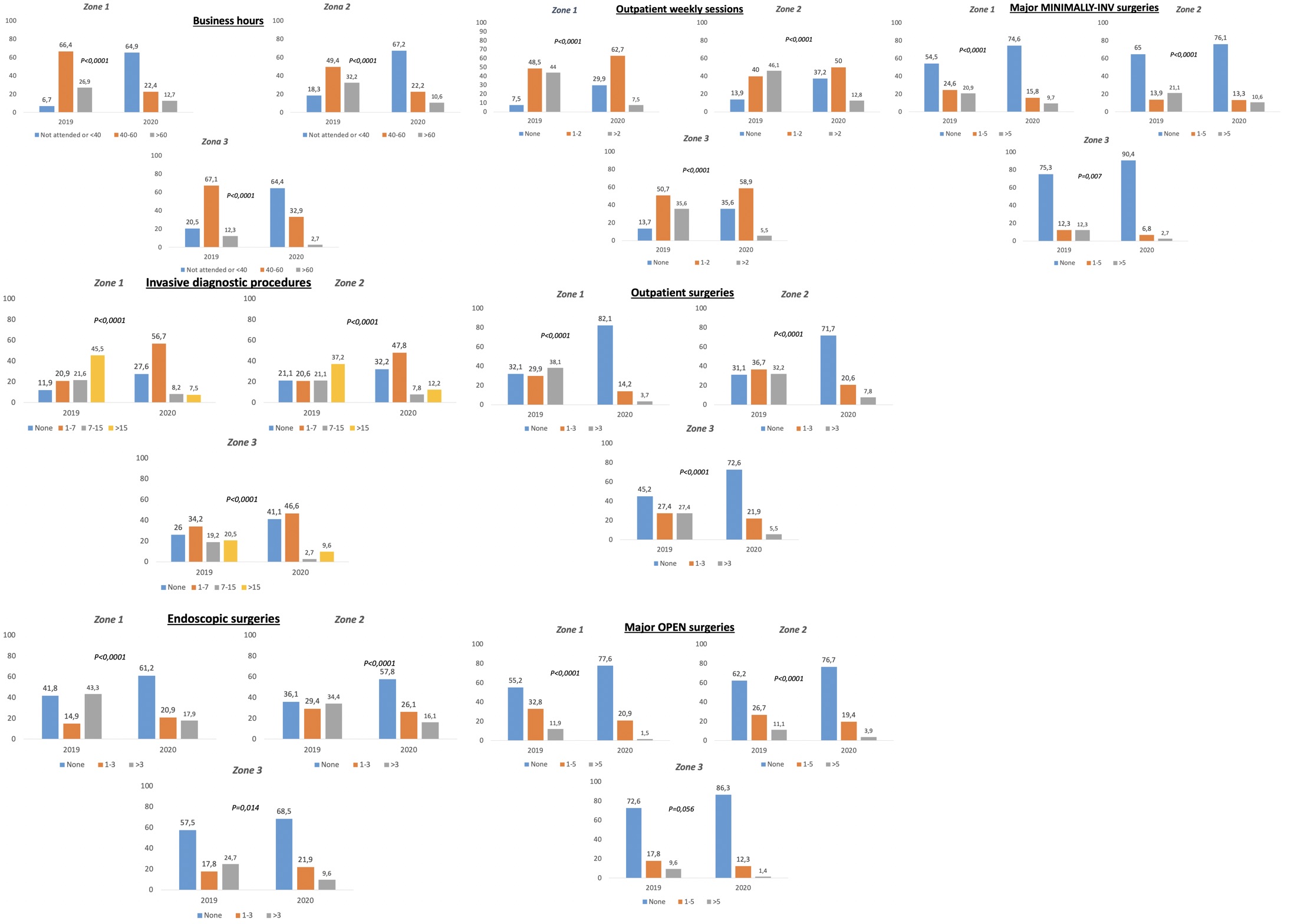

Supplement: Supplementary Figure 4 — Histograms comparing different survey items between March 2020 (the highest outbreak level in Italy) and October or November 2019 (the non-COVID-19 period), categorized on the basis of geographical zones with different numbers of COVID-19 cases (regions of Italy were clustered in three major zones: zone 1 included regions with ≥10,000 cases, zone 2 included regions with between 2,000 and 10,000 cases, and zone 3 included regions with ≤ 2,000 cases). [file Image_4.JPEG]

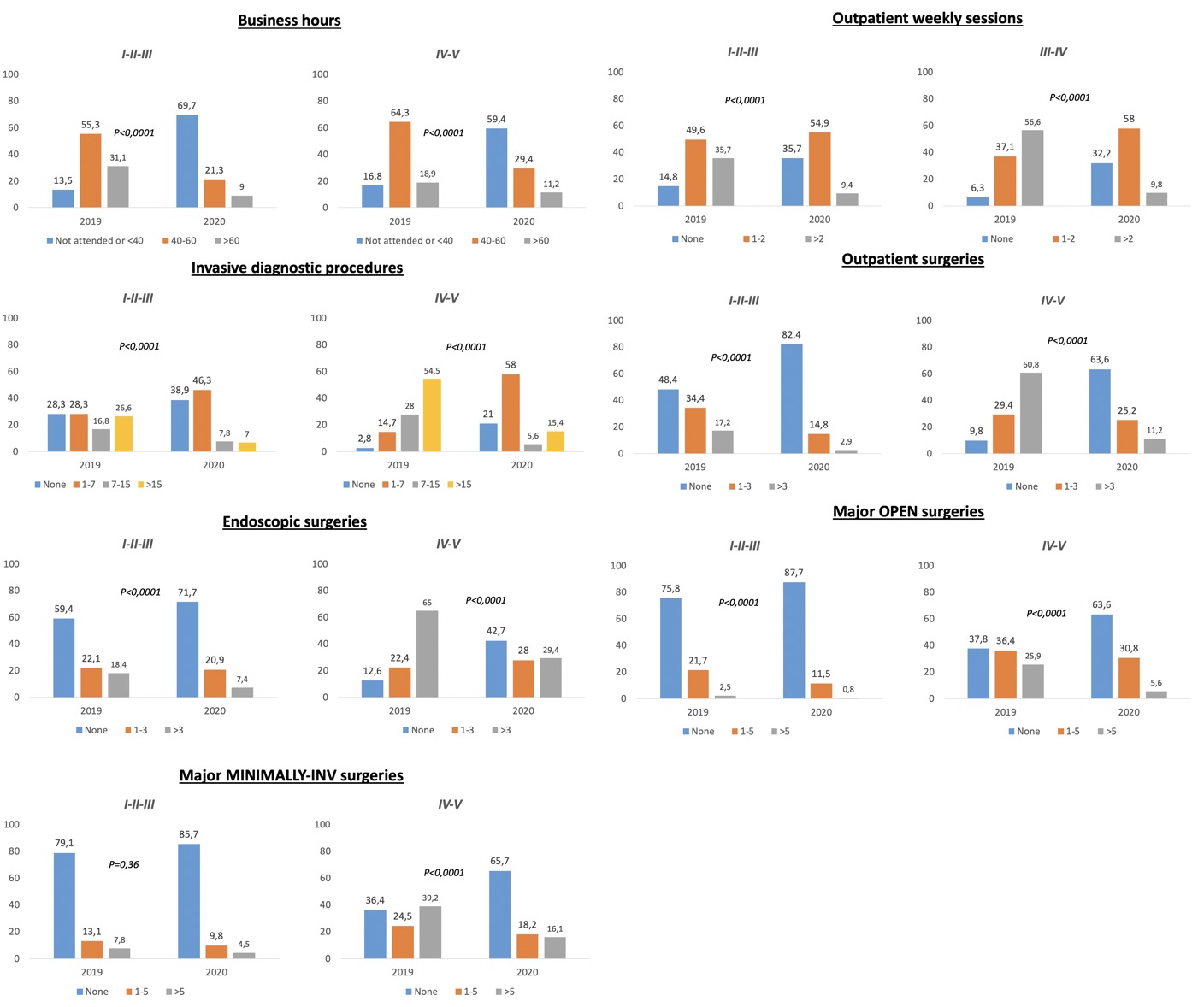

Supplement: Supplementary Figure 5 — Histograms comparing different survey items between March 2020 (the highest outbreak level in Italy) and October or November 2019 (the non-COVID-19 period), categorized on the basis of the year of residency (I-II-III vs. IV-V). [file Image_5.JPEG]
